# Core Palliative Care Competencies for Undergraduate Nursing Education: International Multisite Research Using Online Nominal Group Technique

**DOI:** 10.1177/08258597241244605

**Published:** 2024-04-07

**Authors:** Minna Hökkä, Teija Ravelin, Veerle Coupez, Danny Vereecke, Joanne Brennan, Teodora Mathe, Cornelia Brandstötter, Piret Paal, Daniela Elena Spanu, Nicoleta Mitrea

**Affiliations:** 1 527818 Diaconia University of Applied Sciences, Oulu, Finland; 2 4170 Kajaani University of Applied Sciences, Kajaani, Finland; 3 University of Applied Sciences, Kortrijk, Belgium; 4 602737 European Association for Palliative Care, Vilvoorde, Belgium; 5 113008 University of Transilvania from Brasov, Brasov, Romania; 6 277080 HOSPICE Casa Sperantei, Brasov, Romania; 7 Institute of Palliative Care, 31507 Paracelsus Medical University, Salzburg, Austria; 8 Department of Ethnology, Institute of Cultural Studies, University of Tartu, Estonia

**Keywords:** competence, education, nursing, palliative care, professional competence, Nominal Group Technique

## Abstract

**Background:** Nurses should have appropriate education and required competencies to provide high-quality palliative care. The aim of this international multisite study was to list and evaluate core palliative care competencies that European nurses need to achieve in their education to provide palliative care. **Methods:** The Nominal Group Technique (NGT) was used as a data collection method. NGT meetings were organized in four European countries. Targeted groups of palliative care professionals with diverse contextual and professional backgrounds participated in the NGTs. The research question was: “What are the core competencies in palliative care that need to be achieved during undergraduate nursing education?” Data analysis was done in two stages: grouping the top 10 answers based on similarities and thematic synthesis based on all the ideas produced during the NGTs. **Results:** Palliative care core competencies based on the research were (1) competence in the characteristics of palliative care; (2) competence in decision-making and enabling palliative care; (3) symptom management competence in palliative care; (4) competence in holistic support in palliative care; (5) active person- and family-centered communication competence in palliative care; (6) competence in empathy in palliative care; (7) spiritual competence in palliative care; (8) competence in ethical and legal issues in palliative care; (9) teamwork competence in palliative care; and (10) self-awareness and self-reflection competence in palliative care. **Conclusions:** It was possible to find differences and similarities in the top 10 palliative care core competencies from different countries. Thematic synthesis of all the data showed that there were various competencies needed for nursing students to provide quality palliative care.

## Introduction

The World Health Organization (WHO) defines palliative care as an approach that improves the quality of life of both patients and their families facing problems associated with a life-limiting illness. Palliative care prevents and relieves suffering through early identification, assessment, and treatment of pain and other problems, whether physical, psychosocial, or spiritual.^1^ The WHO and the Council of Europe emphasize that access to palliative care should be considered as a human right and integrated into the healthcare system of every country.^2,3^ To ensure both quality palliative care and the ability to work with community resources, nurses need to be provided with different levels of education and develop appropriate palliative care competencies.^4-9^

Over the past decade, there have been increasing attempts in different countries to include palliative care in nursing curricula through the development of competency frameworks and models^10-16^ Nevertheless, there is still a wide variation in palliative care education between and within European countries, when 56% of the countries from which data was achieved, reported that palliative care is not a mandatory subject in nursing education.^17^ In addition, nursing students have reported a lack of competence^18,19^ and insufficient knowledge about palliative care.^20,21^ Moreover, only half of students in a national survey reported that the education they received on palliative care was sufficient.^19^ Although adequate training is considered a prerequisite for the successful integration of palliative care into health care systems,^8,22^ the extent to which palliative care is integrated into undergraduate nursing education still needs to be developed.^17-25^

When reforming nursing education, it is important that the curricula are evidence-based.^26^ The NursEduPal@Euro project was initiated to improve the quality of palliative care nursing education by enhancing the palliative care competencies of undergraduate nurses and their educators. Competencies in the project were defined as knowledge, skills, values, and attitudes (heightened through self-awareness), that a nurse should possess to successfully perform quality palliative care.^27,28^ As the first step, four countries—Austria, Belgium, Finland, and Romania—set out a process to define those competencies. The aim of this international multisite study was to list and evaluate core palliative care competencies that European nurses need to achieve in their training to provide high-quality palliative care.

## Methods

### Study Design

The data were collected using the Nominal Group Technique (NGT). The NGT is a structured method for obtaining information on a specific topic. It is usually used as a cross-sectional rather than a longitudinal method and is often referred to as a starting point for research.^29^ In previous studies, the NGT has been used to build consensus in nursing research,^30^ to design educational programs and in the identification of required competencies.^29,31,32^

**Figure 1.**
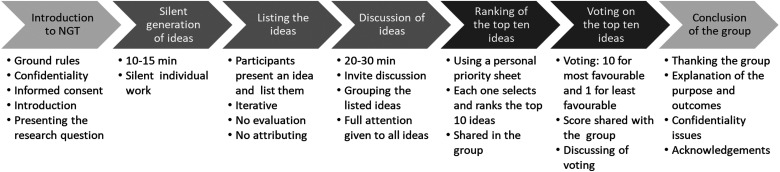
Sequence of steps of a nominal group inquiry (Adapted from Junger and Payne^29^(^p204^)).

The study consists of four phases: (1) preparation and pretesting of the NGT, (2) sampling, (3) group meeting, and (4) data analysis.^29^ The NGT group meeting consists of an introduction, followed by five steps for data collection, and concludes with closing remarks (Figure 1). Typically, participants are welcomed during the introduction and ground rules are established, such as, that all ideas are important. The duration of the meeting is set, informed consent is obtained, and the research question is introduced. In the first step of data collection, participants think silently about the question and develop ideas. Second, the ideas are listed. In the third step, the ideas are discussed in the group. Fourth, the 10 best ideas are ranked. In the fifth step of the data collection, the 10 best ideas are voted on and the result is discussed. At the end of the NGT session, the participants are thanked, and the purpose and results of the study are repeated. Confidentiality requirements are ensured and information about the results of the study is agreed upon.^29^

For this study, the four research teams (see Supplemental Table S1) received online training on the NGT method. Afterward, the teams organized online meetings to pretest all steps of the method. A guideline for the NGT meetings was prepared, including the research question and the size and composition of the NGT groups. Mentimeter, a versatile interactive presentation and polling tool, was chosen as the voting tool. During data collection, Mentimeter enables participants to submit their responses anonymously, providing a secure environment for expressing opinions. During the NGT meeting, Mentimeter was used to vote on the top 10 ideas identified by the participants. Due to the COVID-19 pandemic, the sessions were conducted online. The feasibility and acceptability of adapting NGT meetings to an online format have been demonstrated.^33^

### Setting and Participants

In each participating country, a targeted group of professionals with similarly diverse contextual and professional backgrounds was invited, including nurses (general care, palliative care, home care, elderly care), nurse educators (in palliative care), psychologists, spiritual counselors, physicians, and social workers with experience in palliative care. The aims were (1) to achieve maximum comparability between the NGTs, (2) to obtain a perspective of competencies by including nurses working in different contexts and nurse educators, (3) to obtain a broad view by inviting the multidisciplinary team to define the roles and responsibilities assigned to nurses within the multidisciplinary collaborative framework, and (4) to achieve a broad geographical coverage of the participating countries. An overview of the composition of the four groups is given in Table 1.

**Table 1.** Composition of the NGT Groups in the Different Participating Countries: Professions and Number of Participants.

Country	Nurse	Physician	Nurse educator	Psychologist	Social worker	Spiritual counsellor	Research assistant
Austria	1	1	3	-	-	2	1
Belgium	4	1	1	1	1	1	-
Finland	4	1	1	1	1	1	-
Romania	4	1	1	1	1	1	-

Data on the participant's gender, age, education, and experience in palliative care, as well as the geographical area covered, are summarized in Table 2. The Finnish and Romanian NGT groups were exclusively female, whereas the Belgian and Austrian groups also included men. The average age of the participants ranged from 38 to 49.5 years. Most participants had received formal training in palliative care and had between 1 and 26 years of practical experience in palliative care.

**Table 2.** Overview of the Background Information of the NGT Participants in the Four Countries.

Country	Gender	Average age	Official education in palliative care	Experience in palliative care	Coverage of the country
Austria	1 male 7 females	49,5 years	5/8	Yes: 8/8 Range: 1-20 Average: 11 years	4 of the 9 states
Belgium	3 males 6 females	46,5 years	5/9	Yes: 9/9 Range: 5-26 years Average: 13 years	5 of the 5 Flemish provinces
Finland	9 females	45 years	8/9	Yes: 9/9 Range: 1-22 years Average: 10 years	South, North, West and East of Finland
Romania	9 females	38 years	9/9	Yes: 9/9 Range: 2–24 Average: 12 years	6 of the 8 Romanian regions

### Data Collection, Synthesis, and Analysis

The NGT meetings in each country were organized as two-hour duration sessions between March and May 2021. Each group had two facilitators from the project team, one leading the process and discussion and, the other acting as a scribe.

All group sessions began with a welcome, acknowledgment, and introduction to the steps of the NGT process. Before starting the data collection (see Figure 1), the European experts were presented with the following research question: “What are the core competencies in palliative care that need to be achieved during undergraduate nursing education?” The meetings were held in the national language of each country and one member of each national team translated the results into English. During all stages of the analysis, both English- and non-English-speaking authors of this study discussed the translations and concepts used in the categories to ensure common understanding.

Data analysis was carried out in two stages. First, to compare the results between countries, two independent researchers grouped the top 10 responses based on similarities. Feedback from the workgroups during the data collection process led to the consensus that the top 10 lists from the four countries were not comprehensive. Therefore, in the second stage, all the ideas generated during the four NGT meetings that appeared in at least two countries were retained for analysis and a thematic synthesis was performed.^34^ This involved developing the descriptive themes while remaining faithful to the original expressions and by searching for similarities in the data. While the generation of the descriptive themes was carried out by two researchers, in the subsequent interpretive phase, the members of the research team also participated in the synthesis (Supplemental Table S1).

### Ethical Considerations

The recruited participants were informed in writing in advance that participation in the study was voluntary and that they were free to withdraw the study at any time. If they agreed to participate, the professionals were asked to complete an informed consent form before data collection. In Romania, informed consent was given on paper, in other countries, it was given electronically.

Only the Word documents of the NGT group results and the anonymous voting results were stored and archived in a secure, password-protected digital environment. Participants in the nominal groups were assured of complete anonymity in the event of data publication and analysis, as well as in the dissemination of the obtained results.^35^ Only the research teams had access to the collected information.

The NGT group sessions were recorded with the consent of the participants. Participant's anonymity was promoted by asking them to introduce themselves by using only their first name. The cameras were turned off during the NGT meeting. At the beginning of the NGT session, participants were again informed about the aim of the study and reminded that their participation in the NGT group was voluntary. They were also given the opportunity to ask questions about the study.

## Results

Following the NGT process, country-based, ranked top 10 lists of palliative care core competencies were created. Interestingly, the lists differed between the countries in terms of both content and order of importance (see figures in Table 3). There were also similarities between the countries and 10 areas of competence could be derived from the data (Table 3).

**Table 3.** Result of the First Stage of Data Analysis. Thematic Grouping of the Top 10 Competencies of the Four NGT Groups.

Descriptive themes	Austria	Belgium	Finland	Romania
Symptom management competence in palliative care	Symptom management, non-medicinal measures (8^a^)	Have knowledge of the basic principles of pain management, symptom control, and comfort care (1)	the main principles of treatment/care of key symptoms (including pain) (3) pain management effectively, different forms of pain (8) baseline assessment (holistic) physical, mental, emotional, spiritual, social assessment, uses a validated so-called toolset, for example, outcome assessment and complexity collaborative, integrated palliative care outcome scale model in the assessment (10)	Manages health care issues related to medical maneuvers (eg, oral cavity care, catheter care, wound care, stoma care) (6) Identifies and evaluates physical pain and other symptoms of progressive chronic illnesses (10) Develops the skills of awareness of the nature of suffering on a personal level and at the level of the other person (patient, family, colleague) (7)
Communication competence in palliative care	Communication with patients and relatives, communication skills (6)	Active person-centered listening (includes verbal/non-verbal communication, continued questioning, paraphrasing, without prejudice or preconceptions; both to the patient and to the family/environment) (3) Allowing and accommodating the emotional experience of patient, family and environment (5) Acting in function of the patient's needs (respect, autonomy, temporization, persistent therapy/palliative care, making issues negotiable, solving problems, persisting/insisting in function of the patient) (4) Being empathetic (6)	Interaction skills (2) Patient education skills to educate the patient in palliative care or end of life care and the closest ones (7)	Describes and demonstrates qualities, skills and abilities of verbal and non-verbal communication (3)
Interdisciplinary team work competence in palliative care		Multidisciplinary cooperation and (assertive) communication with other disciplines from the central aim: comfort and quality of life of the patient (8)	In palliative care, several professional groups are needed for offering the best help to the patient and family (9)	Collaborates with the interdisciplinary team to meet the needs of the family from the moment of diagnosis and during the mourning period (1)
Competence of the basic principles of palliative care	Dealing with dying and death (10)	Knowledge of the basic principles of palliative care (4 parts of palliative care (body, social, psycho and emotional, existential), curative versus palliative care, holistic care (2)		Clearly presents the basic values of Palliative Care. (2)
Competence in context and culture sensitivity in palliative care	Intercultural competence (7)	Have context sensitivity (care context of the patient and his family/environment, adaptability to specific settings/situations) (10)	Outlining the care chains for the terminally ill, as well as network skills. Meaning the whole service system (5)	
Ethical and legal competence in palliative care	Ethics decisions at the end of life, legal basics, change of therapy target (goals), desire to die (1) Legal basis (2)			Describes and applies medical ethical principles in clinical practice (5)
Self-reflection competence in palliative care	Self-reflection, dealing with grief (4)	Being able to adopt an open attitude (without prejudging, thinking out of the box) (7) Being self-reflective about one's own values and standards (being able to distinguish between one's own views, values, standards and those of others; being aware of the impact one's own values, standards and views may have on professional practice as a nurse; being able to put one's own views, values, standards aside in function of patient-centered practice (9)		
Advanced care planning competence in palliative care			Knows the importance of a proactive treatment plan right from the start, from which point all palliative and available plans are individually planned to take into account the patient and closest ones (1) Need to understand the goals of care and understand the key concepts related to palliative care, so that all professionals speak the same language (4)	Develops a care plan, together with the patient and family, that respects their wishes and that can be used as a guide in the decision-making process (9)
Evidence-based competence in palliative care	Nursing diagnostics (9) Research (importance!)/gain of knowledge (3)			Identifies and evaluates health care issues (4) Applies clinical judgment based on observation, clinical examination and scientific evidence (health care algorithms, procedures, protocols) to plan and implement appropriate and quality care (8)
Palliative care competence in different patient groups	Palliative care in special patient groups (5)		Identifies a dying patient regardless of disease (6)	

a (x) The number between brackets refers to the ranking of the competence in the original data.

As a result of the grouping of the top 10 competencies, similarities could be found between the four countries. Ten competence areas emerged from the top 10 competence data (Table 3). Only two of the competency areas were defined by all four groups: symptom management competence and communication competence in palliative care. Three of the competencies appeared in three of the NGT groups: interdisciplinary teamwork competence in palliative care (Belgium, Finland, Romania), basic principles of palliative care competence (Austria, Belgium, Romania), and competence in context and cultural sensitivity in palliative care (Belgium, Austria, Finland). Five core competencies were identified by two of the NGT groups: competence in advanced care planning in palliative care (Finland, Romania), ethical competence and evidence-based competence in palliative care (Austria, Romania), competence in self-reflection in palliative care (Austria, Belgium), and competence in palliative care in different patient groups (Austria, Finland).

Each NGT team developed many ideas about essential core competencies in palliative care: Austria: 68; Belgium: 135; Finland: 77; and Romania: 73. As a result of the second stage of data analysis, which included all the ideas appeared in at least two countries, 10 analytical themes were identified, consisting of a total of 42 descriptive themes. These are listed in Table 4. As an example of the analysis, the analytical theme “Competence in the characteristics of palliative care” included aspects such as the philosophy and purpose of palliative care. The descriptive theme *The philosophy of palliative care* included the main principles, foundations, holistic concepts, and values related to palliative care. Underneath some examples of the original data:

*AU Principles and fundamentals (meaning: basics)*

*FI concepts of palliative care*

*BE 4 aspects of palliative care (physical, social, psycho and emotional, existential)*

*RO basic values of Palliative Care.*

**Table 4.** Result of the Thematic Synthesis: Palliative Care Core Competencies that Need to Be Achieved During Undergraduate Nursing Education.

Analytical themes	Descriptive themes
Competence in the characteristics of palliative care	The philosophy of palliative care The purpose of palliative care Palliative care in different patient groups
Competence in decision-making and enabling palliative care	Organizing palliative care Critical evidence-based thinking and decision-making in palliative care Advanced care planning in palliative care Advocacy in palliative care
Symptom management competence in palliative care	Identifying and assessing symptoms in the context of palliative care Principles of symptom control in palliative care Symptom management in palliative care Non-pharmacological symptom management in palliative care Pharmacological symptom management in palliative care Pain management in palliative care
Competence in holistic support in palliative care	Responsiveness and supportiveness to psychosocial needs in palliative care Person-centred supporting when working with palliative patients and those most important to them Culturally sensitive supporting in palliative care Grief and supporting in bereavement during the different phases of the palliative care process
Active person-and family-centred communication competence in palliative care	Open and active verbal and non-verbal communication in palliative care Open and active listening in palliative care Discussion of difficult topics in palliative care The importance of being present in palliative care Open-minded and dignified encountering in palliative care Responsive appropriate interaction in palliative care Encounters with the closest ones to patients in palliative care Communication, and interaction with those most important to patients’ in palliative care
Competence in empathy in palliative care	Empathy in palliative care Empathic communication in palliative care
Spiritual competence in palliative care	Meaning of spirituality in the context of palliative care and its importance to patients Assessment of the spiritual needs of the patients in palliative care Support patients with spiritual needs in the context of palliative care Openness and confidence toward spiritual, religious and existential issues in palliative care
Competence in ethical and legal issues in palliative care	Ethical issues in palliative care and end of life situations Working according to moral and ethical values in palliative care Legislation in palliative care
Teamwork competence in palliative care	Interdisciplinarity in palliative care Cooperation in interdisciplinary palliative care team Active, pro-active and confident communication with other disciplines involved in palliative care Teamwork in palliative care
Self-awareness and self-reflection competence in palliative care	Recognizing and dealing with own emotions arising in palliative care Reflecting own emotions of death and loss Self-reflection concerning values and own actions in palliative care Openness to personal and professional growth

## Discussion

The model proposed by the WHO^26^ highlights six essential components needed for optimal palliative care provision. One of these is education and training for all healthcare professionals providing palliative care. Recent research in European countries shows that poorly organized and uncoordinated education is one of the main barriers that hinder the provision of palliative care for all.^36^ Evidence supporting investment in palliative care shows that it is effective in reducing suffering for patients and families and is cost-effective for integrated healthcare systems.^37^ The need for palliative care is increasing and the need for nurses with appropriate palliative care competencies is imminent.

This study identified top 10 palliative care core competencies for undergraduate nurses in four European countries. Based on the thematic synthesis, a more detailed list of palliative care core competencies was developed. The results show the importance of symptom management, communication, interdisciplinary teamwork, ethical and legal competence, competence in self-reflection, advance care planning (ACP), and knowledge of the basic principles of palliative care. In addition, nurses are expected to be competent in contextual- and cultural sensitivity, in evidence-based care, and in the provision of palliative care to diverse patient groups.

There were geographical differences in the results of the NGT group, which can be partly explained due to differences in education of nurses’ and the expected level of competency in these countries. In Austria, the theoretical training of palliative care for undergraduate nurses covers a wide range of topics and ranges from 21.2 h to 2.5 h. Currently, there is no legal incorporation of palliative care in basic education.^38^ In Finland palliative care has recently been included in the national learning outcomes for undergraduate nursing education and a national curriculum recommendation for palliative care education has been published, but data are not yet available to indicate how these are implemented.^39^ In Belgium, palliative care is not mentioned in the national learning outcomes for nurses. A survey of the Flemish bachelor nursing curricula showed that the time spent on palliative care in different institutions ranged from 2 h to 35 h.^40^ The development of the undergraduate palliative care nursing education in Romania has followed the European recommendations.^7,41^ In 2017, a mandatory palliative care module was included in the curriculum,^42^ although the majority of academics teaching the palliative care modules to nurses are physicians.

An EAPC survey conducted in 2018 indicated that self-care and self-reflection are essential components of palliative care education. Accordingly, all nurses should be equipped with knowledge of self-care, how to increase self-awareness, and why it is essential to reflect before taking any action.^43^ Self-awareness and self-reflection have been identified as core competencies in this study and other published studies and frameworks on palliative care competencies^5,7,13,14^ as well as self-care.^10,11^ Patients die across the healthcare settings and “dying with dignity” which includes patient autonomy, physical and psychological well-being, and spirituality. Spirituality has been identified as an important competency in palliative care in this and earlier studies as also in competency frameworks.^7,10,11,44,45^

Due to demographic changes, nurses in clinical practice are increasingly confronted with vulnerable patients who have difficulty expressing their wishes and autonomy.^46^ Ideally, a collaborative approach between physicians and nurses, together with, active engagement with patients and their families, is essential for making end-of-life decisions. This approach ensures a patient- and family-centred model of care, where decisions are informed by the patient's prognosis, aligned with their expressed wishes, and prioritizing the avoidance of potentially harmful treatments. Competency in communication^47^ and teamwork are essential to achieve this.^48^ Furthermore, it has been demonstrated that education and training in ACP enables nursing staff to discuss, in a timely manner, with the patients how they envision the end of their lives and their end-of-life wishes.^48^ Nurses trained in ACP should be involved in decision-making about life-prolonging interventions.^48^

This study recognized, competencies in symptom management, communication, interdisciplinary teamwork, and in the basic principles of palliative care. These competencies have also been recognized in other competence frameworks.^7,10,11,39^ The EAPC recommendation^7^ suggested that nurses should be able to demonstrate their understanding of the key ethical issues in palliative care and to reflect on their own personal views of life. In relation to contextual competence, the recommendation emphasized the nurses’ competence to understand the needs of the patient and family. Evidence-based competence was highlighted as courage to seek opportunities to develop research awareness and promote professional development. The same aspects were covered in this study. However, palliative care competence for different patient groups in nursing was defined as a competence of nurses at an advanced postgraduate level opposite to this study where this was seen as a competence for all graduating nurses.

A focus on the nurse's role in enabling palliative care and in ACP is not as clearly addressed in the EAPC recommendation^7^ as in this study. These differences may reflect the changing role of nursing as seen in recently published consensus papers which emphasize the essential role of nurses in advocacy and ensuring generalist palliative care for all those in need.^9,49^ As discussed above, the results of this study show that there are similarities and differences in the expected palliative care competencies for undergraduate nurses between countries. Additionally, the findings are largely consistent with previous research and reports, although some differences can be identified.

## Strengths and Limitations

This study has several strengths and limitations. One strength is that the participants in the groups represented widely different nursing contexts, educators, and multidisciplinary team members from different countries. The credibility of this study was strengthened by reporting in detail the sampling, data collection, and analysis process. The clarity of the research question and the NGT process was pretested. The sample included people with different backgrounds and knowledge of palliative care. Therefore, it can be assumed that the study population had knowledge of the phenomenon of interest. Furthermore, dependability was strengthened by presenting tables of all themes identified through grouping and thematic synthesis (Tables 3 and 4). It should also be noted that the researchers repeatedly discussed the synthesis throughout the study. Confirmability was strengthened by focusing on the manifest content during the synthesis, to ensure that the findings represented the views of the professionals.^50^

There are also several limitations in this study. One limitation is that there were no students involved although they could have had important knowledge of the phenomenon of interest. Furthermore, there was no opportunity to return the findings to the professionals for comments.^51^ One possible bias is the study sample, as we only invited experts from four different countries. Regarding the NGT groups maximum comparability between the groups was not achieved. There were some differences in the group compositions.

## Conclusions

By using the NGT method it was possible to identify differences and similarities in the top 10 palliative care core competencies from four different countries. While the phenomenon could not be fully described using only the top 10 competencies, thematic synthesis of all the data occurring in at least two countries showed that there are many competencies needed for nursing students to provide quality palliative care. Both similarities and differences were found when compared to previously published literature. The online NGT method proved to be a good method of collecting data across countries, although the results call for further research on this issue to reach a common consensus on the required palliative care core competencies for undergraduate nursing students.

## Supplemental Material

sj-docx-1-pal-10.1177_08258597241244605 - Supplemental material for Core Palliative Care Competencies for Undergraduate Nursing Education: International Multisite Research Using Online Nominal Group Technique Supplemental material, sj-docx-1-pal-10.1177_08258597241244605 for Core Palliative Care Competencies for Undergraduate Nursing Education: International Multisite Research Using Online Nominal Group Technique by Minna Hökkä, PhD, RN, Teija Ravelin, PhD, RN, Veerle Coupez, MSc, RN, Danny Vereecke, PhD, Joanne Brennan, BSc Physiotherapy, Teodora Mathe, PhD, MSc, APRN, Cornelia Brandstötter, MA, Piret Paa, PhD, MA, BA, Daniela Elena Spanu, PhD, RN, and Nicoleta Mitrea, PhD, MSc, APRN, FAAN in Journal of Palliative Care
