# Supplementary material for: Core Palliative Care Competencies for Undergraduate Nursing Education: International Multisite Research Using Online Nominal Group Technique
Source: J Palliat Care. 2024 Apr 7;39(3):217–26. doi: 10.1177/08258597241244605 (PMC11097607; doi:10.1177/08258597241244605)
Supplement: sj-docx-1-pal-10.1177_08258597241244605 - Supplemental material for Core Palliative Care Competencies for Undergraduate Nursing Education: International Multisite Research Using Online Nominal Group Technique [file sj-docx-1-pal-10.1177_08258597241244605.docx]

Supplementary table 1. Researcher characteristics

| Author (gender) | Credentials,  Occupation at the time of the study, Country | Education on qualitative research | Experience in qualitative research |
| --- | --- | --- | --- |
| MH  (female) | RN (Master’s degree), PhD  Senior Advisor at a University of Applied Science. Finland. | Has completed formal qualitative research study modules at Master’s and PhD levels. | Has conducted qualitative research studies. Has taught qualitative research methods. Has supervised Bachelor’s and Master’s and PhD theses which have used qualitative research methods. |
| TR  (female) | RN  PhD  Senior Lecturer at a University of Applied Sciences. Finland. | Has completed formal qualitative research study modules at Master’s and PhD levels. | Has conducted qualitative research studies. Has taught qualitative research methods. Has supervised Bachelor’s and Master’s theses which have used qualitative research methods. |
| VC  (female) | Master’s degree in Nursing. Belgium. | Has completed formal qualitative research studies in Master’s level. | Has conducted qualitative research studies. Has supervised Bachelor’s and Master’s theses which have used qualitative research methods |
| DV  (female) | Master’s degree in Biology, PhD in Biotechnology. Belgium. | Has completed optional qualitative research study modules at basic level | Has conducted qualitative research studies. Has supervised Bachelor’s and Master’s theses which have used qualitative research methods |
| JB  (female) | BSc, Project officer. Ireland. | Has completed formal qualitative research study modules at Master’s level | Has conducted qualitative research. |
| TM  (female) | APRN (Master’s degree), PhD. Romania. | Has completed formal qualitative research study modules at Master’s and PhD levels. | Has conducted qualitative research studies. |
| CB  (female) | Master’s degree in science of education  Research assistant at a University of Medicine. Austria. | Has completed formal qualitative research study modules at Master’s levels. | Has conducted qualitative research studies. Has taught qualitative research methods. |
| PP  (female) | Private lecturer in theory of nursing science (palliative care), Doctoral degree in philosophy. Austria. | Has completed formal qualitative research study modules at all levels. | Has conducted qualitative research studies. Has taught qualitative research methods.  Has supervised Bachelor’s and Master’s theses and Doctoral theses respectively. |
| DES  (female) | RN (Master’s degree), PhD’s. Romania. | Has completed formal qualitative research study modules at Master’s and PhD levels. | Has conducted qualitative research studies. |
| NM  (female) | APRN (Specialty Program in PC and Master’s degree in PC), PhD  Senior Advisor on PC for the Ministry of Health  Senior lecturer at the University of Medicine – Nursing Division. Romania. | Has completed formal qualitative research study modules at Master’s and PhD levels. | Has conducted qualitative research studies. Has taught qualitative research methods. Has supervised Bachelor’s, Master’s and PhD theses which have used qualitative research methods |
